# High-permittivity thin dielectric pad improves peripheral non-contrast MRA at 3T

**DOI:** 10.1186/1532-429X-16-S1-P166

**Published:** 2014-01-16

**Authors:** Marc D Lindley, Daniel Kim, Glen Morrell, Marta E Heilbrun, Pippa Storey, Christopher Hanrahan, Vivian S Lee

**Affiliations:** 1 UCAIR, Radiology, University of Utah, Salt Lake City, Utah, USA; 2 Radiology, New York University, New York, New York, USA

## Background

Non-contrast magnetic resonance angiography (NC-MRA) is an alternative diagnostic tool for assessment of peripheral vascular disease in patients with impaired kidney function. While peripheral NC-MRA based on subtraction of two turbo-spin-echo acquisitions may benefit from increased signal-to-noise ratio (SNR) at 3T, it also suffers from signal loss in the right femoral artery due to B1 inhomogeneities[1], which can be minimized using high-permittivity dielectric pads[2]. The purpose of this study was to utilize high-permittivity dielectric pad to reduce NC-MRA signal loss associated with B1 inhomogeneity.

## Methods

**Figure 1:**
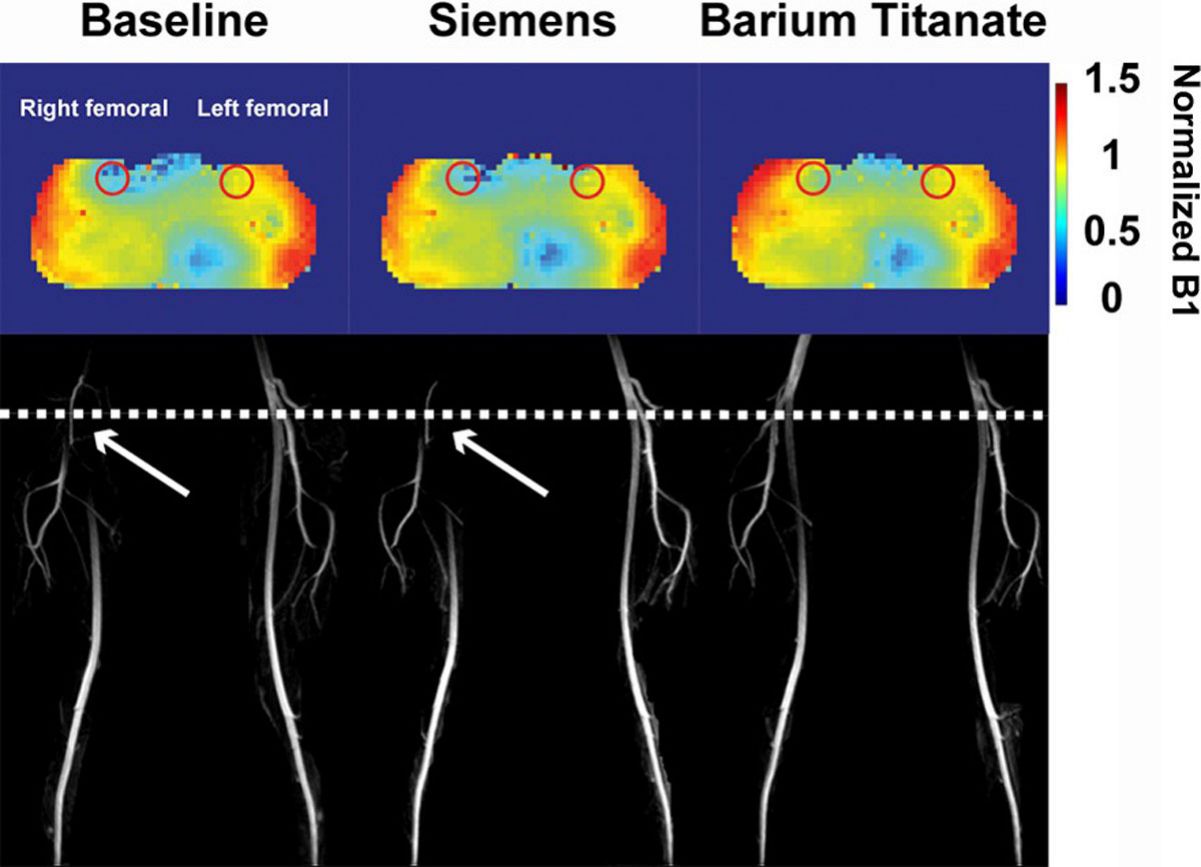
**(Top row) Normalized B1 maps in the axial plane that cross-sections at the common femoral arteries and (bottom row) NC-MRA MIPs: (left column) without pad, (middle column) with commercial pad, and (right column) with high-permittivity pad**. White dotted line corresponds to the axial plane of the B1 map. White arrows point to signal loss in the right femoral artery due to B1 inhomogeneity.

Six healthy volunteers were imaged at 3T(Tim_Trio, Siemens) to compare the following NC-MRA acquisitions with spatial resolution = 1.5 × 1.6 × 2.0 mm and scan time = 3 min: without pad, with commercially available bulky dielectric pad(37 × 25 × 5 cm), and with high-permittivity thin dielectric pad (barium titanate, 38 × 20 × 2 cm), which is 60% thinner than the commercial pad. For details on the MRA protocol, see reference[3]. For each MRA acquisition, we also acquired a B1 map (see reference[4] for more details) in the axial plane to cross-section the common femoral arteries. For quantitative analysis, we calculated apparent contrast-to-noise ratio(CNR) of the left(control) and right common femoral arteries, where apparent CNR is defined as(SIartery-SIbackground_Tissue)/noise. Given that the three acquisitions used identical imaging parameters, except for the dielectric pad, we used the same noise value for CNR comparison for each subject. The mean normalized B1 encircling the left and right common femoral arteries was measured(see Figure 1). ANOVA was used to compare the three CNR groups(with Bonferroni correction to compare each pair). Images were graded by three radiologists in consensus on a Likert scale 1-5(worst-best) for conspicuity of common femoral arteries. Kruskal-Wallis test was used to compare the three conspicuity scores(with Bonferroni correction to compare each pair).

## Results

**Figure 2:**
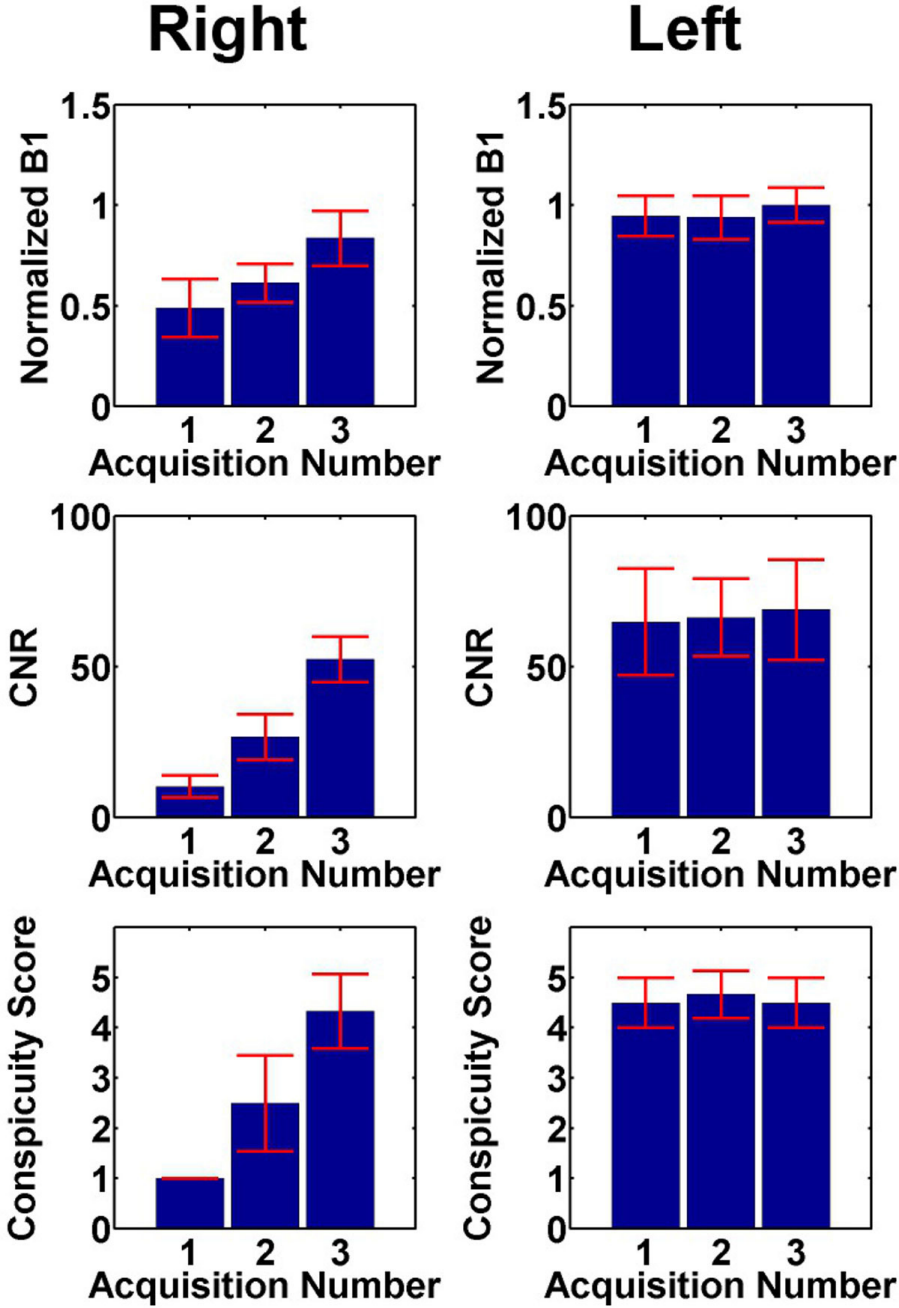
**(Top row) Normalized B1 encircling the right and left femoral arteries**. (Middle row) Apparent CNR in the right and left common femoral arteries. (Bottom row) Normalized B1 encircling the right and left femoral arteries. Values were obtained at the bifurcation point of the deep and superficial femoral arteries. (Bottom row) Conspicuity score graded by three radiologists in consensus. Acquisition number 1: baseline, acquisition 2: commercial pad, acquisition 3: high-permittivity pad.

Compared with baseline and commercial-dielectric-pad acquisitions, high-permittivity-dielectric pad acquisition minimized signal loss in right femoral artery (Figure 1, see B1 map). Over 6 subjects(Figure 2), the mean normalized B1, CNR, and conspicuity score in the left common femoral artery were not different(p > 0.5). In contrast, the mean normalized B1, CNR, and conspicuity score in the right common femoral artery were significantly better with high-permittivity pad acquisitions than baseline and commercial pad acquisitions(p < 0.001).

## Conclusions

Our study shows that NC-MRA signal loss in the right common femoral artery at 3T can be minimized through the use of high-permittivity dielectric pad. This B1 correction allows for the signal loss from the inhomogeneities to be corrected and the common femoral artery to be seen in NC-MRA images.

## Funding

NIH (5R01 HL092439) and Ben B. and Iris M. Margolis Foundation.
